# Sex differences in the genetic and causal relationships between depression, smoking, and alcohol use: the role of socioeconomic status

**DOI:** 10.1017/S0033291726103195

**Published:** 2026-03-02

**Authors:** Jihua Hu, Katrina Grasby, Brittany Mitchell

**Affiliations:** 1 School of Biomedical Sciences, The University of Queensland, Brisbane, QLD, Australia; 2 Brain and Mental Health Program, QIMR Berghofer Medical Research Institute, Brisbane, QLD, Australia

**Keywords:** depression, drinking, genetics, genome-wide association study, mendelian randomization, sex differences, sex-linked, smoking, socioeconomic status

## Abstract

Major depressive disorder (MDD), smoking, and drinking frequently co-occur, with evidence suggesting these relationships may differ by sex. However, the direction of causality and the extent of sex-specific associations remain unclear. We investigated sex-specific genetic relationships between MDD and substance use phenotypes using genome-wide association studies (GWAS) from the UK Biobank and publicly available sex-stratified GWAS for MDD and problematic alcohol use (PAU). Causal effects were assessed using bidirectional, sex-stratified Mendelian randomization (MR). We further applied multivariable MR (MVMR) to evaluate the influence of socioeconomic status (SES). Genetic correlation analyses indicated significant shared genetic architecture between MDD and all substance use traits in sex-combined GWAS. In sex-specific analyses, the correlation between cigarettes per day and MDD was significantly stronger in females, and drinks per week were correlated with MDD only in females. MR analyses showed that genetic liability to MDD increased the risk of smoking initiation and PAU in females, and was associated with reduced alcohol drinking frequency in males. In contrast, no tested substance use trait showed evidence of a causal effect on MDD in either sex. MVMR adjusting for SES attenuated the association between MDD and smoking initiation. The effect on PAU in females remained. In males, the negative association between MDD and drinking frequency became non-significant after SES adjustment. These findings reveal sex-specific genetic and causal relationships between smoking, drinking, and MDD, and highlight the role of SES as a potential confounder. Incorporating sex and socioeconomic context is critical when examining these associations.

## Introduction

Major depressive disorder (MDD), also known as depression, is one of the most common psychiatric conditions in the world and a leading cause of disability (Friedrich, 2017). Depression and substance use frequently co-occur and influence each other (Calarco & Lobo, 2021). Tobacco and alcohol use are particularly common among individuals with MDD (Han et al., 2022). Individuals with MDD tend to smoke more heavily and suffer worse health outcomes than those without MDD (Lê Cook et al., 2014), and smoking itself is linked to a higher risk of developing MDD (Fluharty, Taylor, Grabski, & Munafò, 2017). Alcohol use disorder (AUD) is also highly comorbid with depression (Brière et al., 2014). People with AUD are approximately 2.3 times more likely to have MDD (McHugh & Weiss, 2019). These conditions co-occur more frequently than expected by chance (Holma, Holma, & Isometsä, 2020) and are associated with more severe clinical outcomes (Swendsen & Merikangas, 2000).

Although this comorbidity is well established, its underlying nature remains elusive and potentially bidirectional. Substance use may have a causal effect on MDD, as it can lead to social, interpersonal, and health-related challenges (Swendsen & Merikangas, 2000), and longitudinal studies found that early substance use predicts later onset of depression (Brook et al., 2002). Conversely, individuals with depression may use substances to self-medicate (Khantzian, 1997). About 22–24% of those with mood disorders report using substances to relieve their symptoms (Turner, Mota, Bolton, & Sareen, 2018), and some developed substance use disorders (Kuo, Gardner, Kendler, & Prescott, 2006; Wolitzky-Taylor et al., 2012). A third possibility is that shared genetic or environmental risk factors. Early life adversity, such as childhood trauma or family dysfunction, has been shown to increase the risk of both (Dube et al., 2003; Enoch, 2011), and genome-wide association studies also find significant genetic correlations between depression and smoking or drinking (Johnson et al., 2025; H. Zhou et al., 2023). Some confounding factors such as socioeconomic status (SES) might also contribute to the comorbidity, as individuals with lower SES are at higher risk for both conditions (Collins, 2016; Freeman et al., 2016), and related traits such as educational attainment showed negative genetic correlations with both depression and substance use (Kember et al., 2023; Wray et al., 2018). As no single model fully explains the comorbidity, it is likely that multiple pathways contribute simultaneously (Swendsen & Merikangas, 2000).

Historically, most evidence on comorbidity comes from retrospective and longitudinal studies, but these approaches yield mixed findings likely due to recall bias and challenges of disentangling complex, bidirectional relationships in observational data (McHugh & Weiss, 2019). Genetic methods like Mendelian randomization (MR) offer an alternative strategy for testing causality with evidence for a causal effect of smoking initiation on MDD, and weaker support for the reverse direction (Wootton et al., 2020). MDD has been found to increase the risk of alcohol dependence without a reverse effect (Polimanti et al., 2019). These studies revealed potential causal links between MDD and smoking and alcohol use, but generally assume a uniform effect across sexes.

Sex is an essential factor to be considered because the burden of comorbidity of depression and substance use is not equally distributed. Females are nearly twice as likely as males to experience MDD (Salk, Hyde, & Abramson, 2017), whereas males report higher rates of smoking and alcohol use (Peters, Huxley, & Woodward, 2014; A. M. White, 2020). Beyond prevalence, sex-specific patterns emerge in the course and consequences. Females with MDD may progress more rapidly from substance use to dependence and experience more severe symptoms related to smoking and alcohol (Boykoff et al., 2010; Smith et al., 2016). In alcohol use, longitudinal studies have suggested a sex-dependent temporal relationship: females more often develop MDD before AUD, while AUD typically precedes MDD in males (Hanna & Grant, 1997). Additionally, depressive symptoms appear to be a stronger predictor of alcohol use escalation among females than males (Foster, Hicks, Iacono, & McGue, 2015; Moscato et al., 1997).

These findings suggest that the relationship between depression and substance use may differ between males and females. Given that smoking, drinking, and depression are all influenced by genetics (Shadrina, Bondarenko, & Slominsky, 2018; Verhulst, Neale, & Kendler, 2015; Vink, Willemsen, & Boomsma, 2005), exploring these relationships genetically offers a chance to understand potential sex-specific mechanisms. In this study, we use ‘sex-specific’ to refer to genetic associations estimated separately in males and females, whether the loci are unique to one sex or shared but differ in effect size. Twin studies find that smoking initiation is more heritable in females, while smoking persistence or dependence is more heritable in males (Li, Cheng, Ma, & Swan, 2003). Similarly, a large twin study found significantly higher heritability of MDD in women (Kendler, Gatz, Gardner, & Pedersen, 2006). Genome-wide association studies (GWAS) have shown that the genetic correlation between male and female problematic alcohol use is significantly less than one (H. Zhou et al., 2023), suggesting the presence of sex-specific genetic effects. A population-based twin study found that the genetic and environmental overlap between MDD and AUD was significant only within sexes, not across sexes, implying that the mechanisms linking these disorders may differ between men and women (Prescott, Aggen, & Kendler, 2000).

Despite evidence of sex differences in depression and substance use, it remains unclear whether the genetic pathways linking these traits also differ between sexes. Using sex-stratified GWAS data on major depressive disorder, smoking behaviors, and alcohol-related traits, this study estimates specific genetic correlations and bidirectional causal effects using MR to determine whether the causal relationship between depression and substance use differs by sex.

## Methods

**Figure 1.**
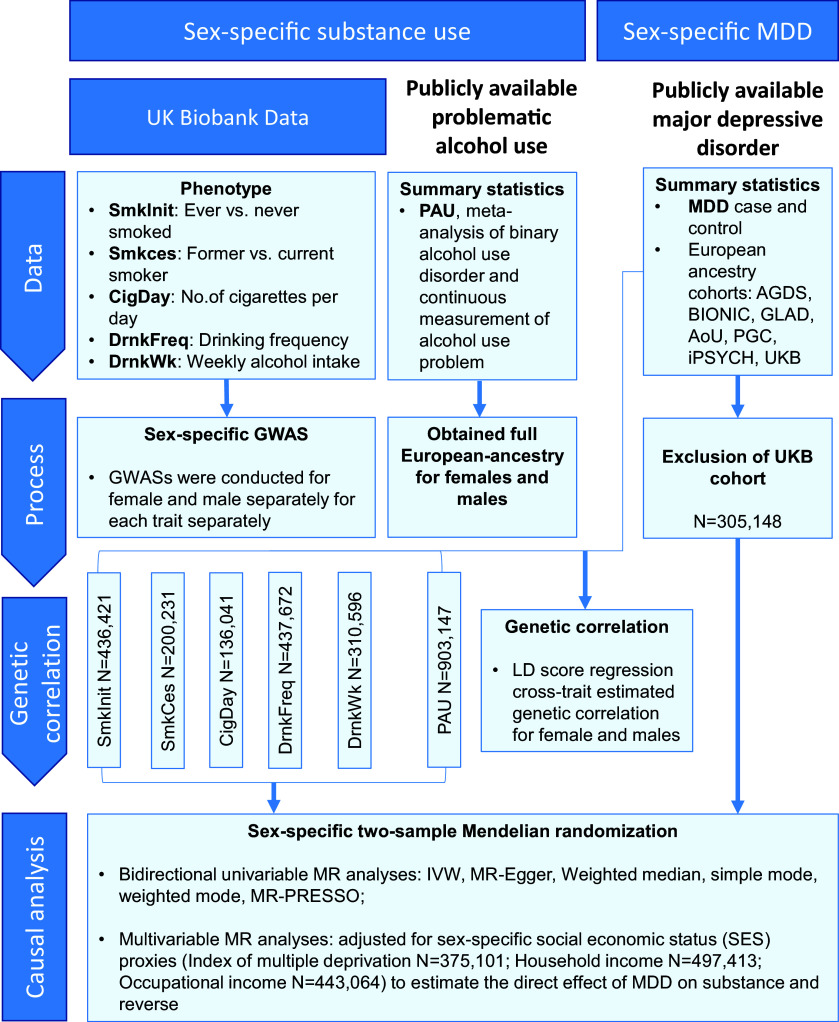
Flowchart of the study design. The study investigated the bidirectional causal relationships between major depressive disorder (MDD) and substance use traits, stratified by sex. Substance use traits included smoking initiation (SmkInit), smoking cessation (SmkCes), cigarettes per day (CigDay), drinking frequency (DrnkFreq), drinks per week (DrnkWk), and problematic alcohol use (PAU). Socioeconomic status (SES) was included as an additional exposure in multivariable Mendelian randomization analyses.

We conducted a bidirectional, sex-stratified Mendelian randomization study to examine causal relationships between depression and substance use traits, with socioeconomic status included in multivariable models. In all analyses, the exposure refers to genetically predicted liability to each trait rather than the observed behavioral measure. This study followed the STROBE-MR guidelines (Supplementary Table S1). An overview of the study workflow is presented in Figure 1.

### Data sources

#### Depression

Summary statistics for depression were obtained from sex-stratified meta-analyses of GWAS conducted in individuals of European ancestry, on a total of 289,992 females and 196,990 males (Thomas et al., 2025). Independent genome-wide significant variants were identified from these meta-analyses. To avoid bias in the MR analysis due to significant sample overlap between the exposure and the outcome from the UK Biobank cohort (UKB), the effect estimates for MR were selected from a version of the sex-stratified meta-analyses that excluded UKB data. The UKB excluded version was generated using a leave-one-out meta-analysis in which the UKB cohort was removed (46,194 female and 22,608 male cases were excluded), and the remaining contributing cohorts were meta-analyzed. Using separate datasets for instrument discovery and effect estimation adopts the key idea of three-sample MR (Burgess et al., 2019; Zhao et al., 2020).

### Problematic alcohol use (PAU)

We used publicly available summary statistics for problematic alcohol use (PAU) from a recent large meta-analysis comprising 11 cohorts and 903,147 individuals (143,198 females and 496,548 males) (H. Zhou et al., 2023). PAU consists of data from clinical diagnoses of alcohol use disorder (AUD) and self-reported problematic drinking behavior. Potential overlap between depression and PAU cohorts was limited, accounting for at most 5% of the PAU sample (PGC, QIMR AGDS, iPSYCH1/2).

### Smoking and alcohol phenotypes in the UK Biobank

We generated these results by conducting sex-specific GWASs using individual-level data from the UK Biobank. Participants included in these analyses were of White British ancestry (MacGregor et al., 2018), with exclusions applied to individuals with mismatched self-reported and genetic sex or who had withdrawn consent.

Smoking behavior was assessed using three phenotypes. Smoking initiation (SmkInit) was defined as having ever smoked versus never smoked, based on the UKB field 20116. Smoking cessation (SmkCes) was defined as former versus current smoker (UKB field 20116). Cigarettes per day (CigDay) was derived from fields 3456 and 2887 for current and former smokers, respectively.

Alcohol use was captured using two traits: drinking frequency (DrnkFreq) and weekly alcohol intake (DrnkWk). Drinking frequency (UKB field 1558) was measured on a scale ranging from ‘Never’ to ‘Daily or almost daily’. Responses were reverse-coded to a 0–5 scale, with higher values indicating more frequent alcohol use.

Weekly alcohol intake (DrnkWk) was based on self-reported consumption of wine, beer, spirits, and fortified wine (fields 1568–1608). Beverage quantities were converted into standardized alcohol units, assuming comparable serving sizes and alcohol content as in previous studies of this trait (Marees et al., 2020). DrnkWk was log-transformed, and the effects of age and weight were regressed out separately in males and females as described in previous work (Clarke et al., 2017). The resulting residuals were used as the phenotype in our sex-specific GWAS.

### Socioeconomic status (SES)

We adopted multiple measurements to capture SES, including the index of multiple deprivation (IMD), household income, and occupational income. IMD scores are based on multiple domains, including crime, education, employment, health, housing, income, and living environment scores, and have been used as proxies for SES (Cadar et al., 2018). To ensure interpretability where higher values corresponded to higher SES, IMD scores were multiplied inversely in this study. Household income reflects the combined incomes of all household members and therefore captures both individual and household-level socioeconomic factors. Occupational income was obtained from national labor statistics reporting average earnings, capturing not only income potential but also educational requirements (Kweon et al., 2025). Sex-specific GWASs for IMD were derived from the UK Biobank (field 26410), and the sex-specific GWASs for household and occupational income were obtained from a publicly available large-scale meta-analysis (Kweon et al., 2025).

Table 1 summarizes the European-ancestry GWAS datasets used, including sample sizes, data sources, and the number of SNP instruments included in downstream MR analyses.

**Table 1.** Overview of GWAS datasets used for instrument selection and MR analyses

Phenotype	Sex	Source	N	N cases	N controls	number of IVs
SmkInit	Combined	UK Biobank	436,421	200,231	236,190	95
	Female		236,222	97,195	139,027	21
	Male		200,199	103,036	97,163	27
SmkCes	Combined		200,231	155,024	45,207	5
	Female		97,195	76,260	20,935	2
	Male		103,036	78,764	24,272	2
CigDay	Combined		136,041	/	/	7
	Female		65,667	/	/	5
	Male		70,374	/	/	3
DrnkFreq	Combined		437,672	/	/	63
	Female		236,902	/	/	21
	Male		200,770	/	/	12
DrnkWk	Combined		310,596	/	/	26
	Female		163,221	/	/	5
	Male		169,941	/	/	10
PAU (Zhou et al., 2023)	Combined	MVP, FinnGen, UKB, PGC, AGDS, QIMR TWINS, QIMR GBP, iPSYCH, YP3	903,147	/	/	63
	Female		143,198	/	/	3
	Male		496,548	/	/	32
Negative IMD (transformed by multiply −1)	Combined	UK Biobank	375,101	/	/	26
	Female		202,770	/	/	5
	Male		172,331	/	/	4
Household income (Kweon et al., 2025)	Combined	ALSPAC, CoLaus, Croatia - Korcula, 1958, AddHealth, EGCUT, ELSA, FTC, GFG, GS, HRS, HUNT, iPSYCH, KORA, Lifelines, MCTFR, MoBa, NEO, NTR, QIMR, RS, SHIP, STR, UKB, UKHLS, WLS	497,413	/	/	40
	Female		273,577	/	/	12
	Male		223,836	/	/	10
Occupational income (Kweon et al., 2025)	Combined		443,064	/	/	62
	Female		252,546	/	/	36
	Male		190,518	/	/	8
MDD (Thomas et al., 2025)	Combined	AGDS, AoU, Bionic, PGC, iPSYCH, GLAD, UKB	486,982	195,276	291,706	16
	Female		289,992	130,471	159,521	16
	Male		196,990	64,805	132,185	6
MDD UKB excluded	Combined	AGDS, AoU, Bionic, PGC, iPSYCH, GLAD	305,148	126,474	178,674	16
	Female		187,282	84,277	103,005	16
	Male		117,866	42,197	75,669	6

*Note:* The following values represent (unscaled) means for continuous phenotypes derived from UK Biobank data. CigDay: combined = 18.17; female = 15.99; male = 20.21. DrnkFreq: combined = 3.05; female = 2.80; male = 3.34. DrnkWk (After the unit conversion between different types of alcohol), combined = 19.97; female = 14.45; male = 25.30. IMD (raw, higher values indicate greater deprivation): combined = 17.68; female = 17.41; male =18.00.

### Genome-wide association analyses

To facilitate comparison with existing literature, we conducted both sex-combined and sex-specific GWASs using REGENIE v2.2.4 (Mbatchou et al., 2021) for each exposure phenotype. Autosomal and X chromosome variants were included, with male X chromosome genotypes coded as 0/2 to assume full dosage compensation. Logistic regression was used for binary traits (SmkInit and SmkCes), and linear regression was used for continuous traits (CigDay, DrnkFreq, DrnkWk, and IMD). In sex-combined GWASs, models were adjusted for sex and the first four genetic principal components (PCs), while sex-specific GWASs included only the first four PCs. We evaluated potential confounding from population stratification using genomic control lambda and LD Score regression (LDSC) intercepts using LDSC (v1.0.1) (Bulik-Sullivan et al., 2015).

### Heritability and genetic correlation

SNP-based heritability of each trait and pairwise genetic correlations between substance use and depression were estimated using LDSC (v1.0.1) (Bulik-Sullivan et al., 2015). Differences in genetic correlation by sex were assessed using a Z-test for independent correlation estimates, and the resulting *p*-values were adjusted for multiple testing (Benjamini & Hochberg, 1995).

### Univariable Mendelian randomization

Mendelian randomization (MR) is based on three core assumptions: (1) the genetic instruments are robustly associated with the exposure (relevance); (2) the instruments are independent of confounders of the exposure-outcome relationship (independence); and (3) the instruments influence the outcome only through the exposure and not via alternative pathways (exclusion restriction or no horizontal pleiotropy).

Independent instrumental variables (IVs) were selected for each exposure at genome-wide significance (*p* < 5 × 10^−8^) and clumped for linkage disequilibrium (r^2^ < 0.01) using PLINK v1.90 (Purcell et al., 2007). Instrument strength was assessed by calculating the F-statistic for each SNP using the formula F = β^2^/SE^2^ (Pierce, Ahsan, & Vanderweele, 2011) and then averaging across SNPs to obtain an overall F-statistic for each exposure.

To assess potential violation of the exclusion restriction assumption, we conducted a series of sensitivity analyses alongside the primary inverse-variance weighted (IVW) MR approach. These included MR-Egger regression, weighted median, weighted mode, simple mode, MR-PRESSO (global test and outlier correction), and Cochran’s Q statistic for heterogeneity. For the analyses between PAU and MDD, which may involve overlapping participants, we additionally applied MRlap to obtain causal estimates that accounting for sample overlap. We interpreted consistent effect estimates across methods, a non-significant MR-Egger intercept, and the absence of influential outliers identified by MR-PRESSO as supportive of the MR assumptions being reasonably satisfied.

Analyses were conducted in R (v4.2.0) using the TwoSampleMR (v0.6.15) (Hemani et al., 2018), MR-PRESSO (v1.0) (Verbanck, Chen, Neale, & Do, 2018), and MRlap (v0.0.3) (Mounier & Kutalik, 2023) packages. False discovery rate (FDR) correction (Benjamini & Hochberg, 1995) was applied to *p*-values from IVW, simple mode, weighted median, and weighted mode methods *p*-values, within each sex and each causal direction.

### Multivariable Mendelian randomization

To account for potential confounding by SES, we conducted multivariable Mendelian randomization (MVMR) analyses, which estimate the direct causal effect of one exposure on an outcome while adjusting for additional exposures (Sanderson, 2021). SES proxies were each included as a second exposure variable alongside either MDD or substance use traits. MVMR analyses were performed using the MVMR (v0.4.1) R package (Sanderson, Spiller, & Bowden, 2021). Instrument strength was evaluated using conditional F-statistics. We applied MVMR bidirectionally to assess the direct effects of MDD and SES on substance use traits, and the direct effects of substance use traits and SES on MDD. FDR correction (Benjamini & Hochberg, 1995) was applied to all MVMR *p*-values within each sex and causal direction for each SES measurement.

## Results

### Sex-specific genome-wide associations

Sex-specific GWASs conducted in the UK Biobank were evaluated for quality, with LDSC intercepts showing only modest inflation across all traits (Supplementary Table S2), indicating that population stratification and other confounders were well controlled. Furthermore, we assessed the genetic correlations between our UKB-based sex-combined GWASs and the largest available public summary statistics from the GSCAN consortium (Saunders et al., 2022). All traits demonstrated strong and significant genetic correlations with their corresponding GSCAN counterparts (genetic correlation range: 0.74 to 0.99; all *p* < 1 × 10^−300^) (Supplementary Table S3), supporting the validity and consistency of our GWAS results. Manhattan plots for each sex-specific GWAS in the UKB are provided in Supplementary Figures (Figures S1–S6).

### Sex-specific genetic correlation

**Figure 2.**
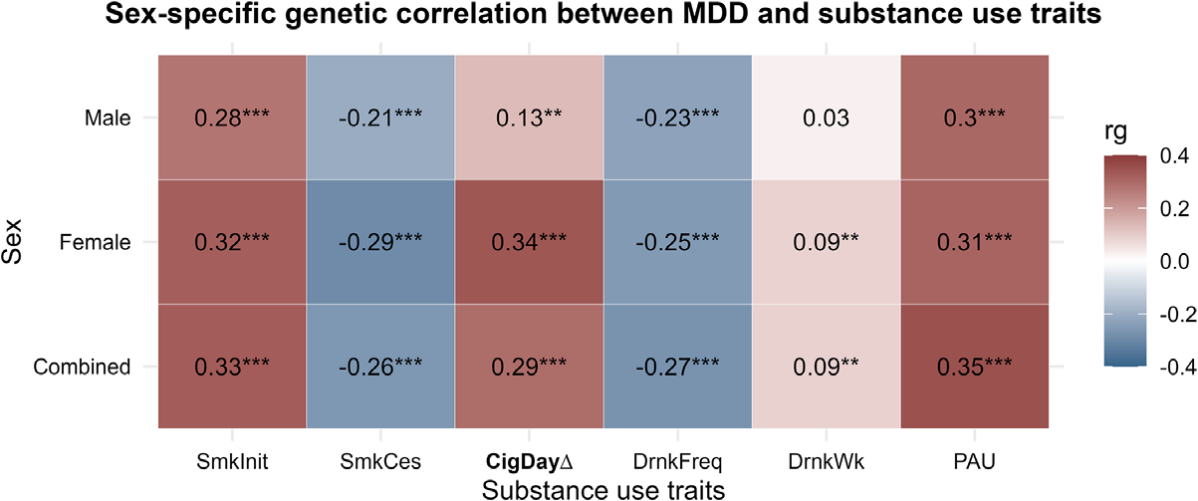
Heatmap showing sex-specific genetic correlations between substance use behavior and major depressive disorder (MDD). Smoking initiation is abbreviated as SmkInit, smoking cessation as SmkCes, cigarettes per day as CigDay, drinking frequency as DrnkFreq, drinks per week as DrnkWk, and problematic alcohol use as PAU. * indicates FDR-adjusted *p* < 0.05, ** indicates FDR adjusted *p* < 0.01, and *** indicates FDR adjusted *p* < 0.001. ∆ indicates a significant difference in genetic correlation between females and males based on a Z-test.

Overall, all substance use traits were significantly genetically correlated with our sex-combined GWAS of depression (Figure 2). These correlations were largely consistent in the sex-stratified analyses, with the exception of drinks per week in males, which was not significantly correlated with male-specific MDD (rg = 0.03, SE = 0.05, P = 0.48). In contrast, drinks per week showed a significant correlation with depression in females (rg = 0.14, SE = 0.05, FDR-adjusted P = 7.2 × 10^−3^), but the correlation did not differ significantly between sexes (Z = 1.17, FDR-adjusted P = 0.49).

Notably, the genetic correlation between cigarettes per day and depression was significantly stronger in females (rg = 0.34, SE = 0.05) than in males (rg = 0.13, SE = 0.04), with a Z-difference of 3.49 (FDR adjusted P = 7.08 × 10^−2^). These findings support the presence of shared genetic architecture between substance use and MDD and suggest differences between females and males that warrant further exploration.

### Sex-combined bidirectional MR and MVMR of MDD and substance use

**Figure 3.**
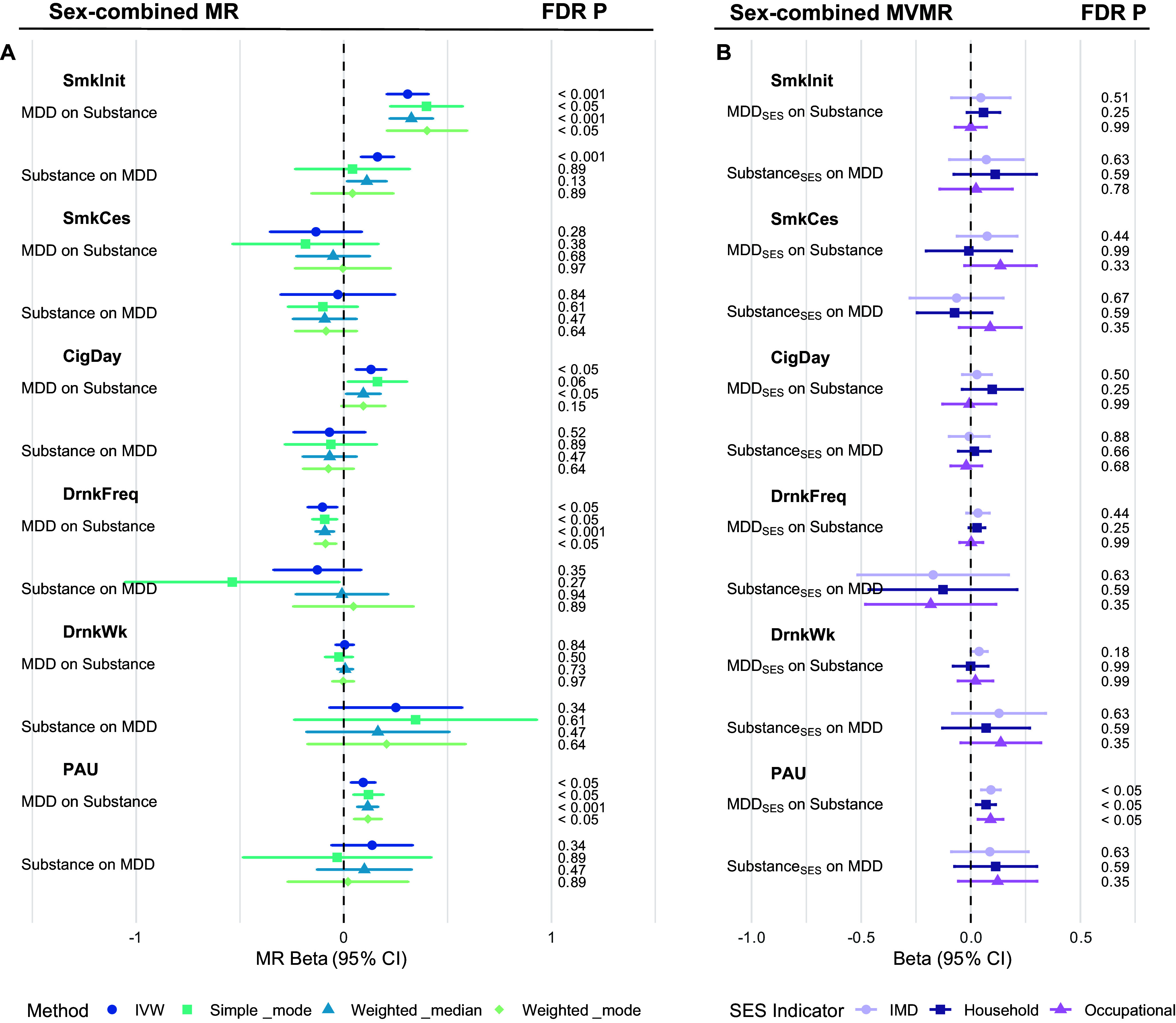
Forest plots of bidirectional Mendelian randomization (MR) and multivariable MR (MVMR) estimates of the causal relationship between major depressive disorder (MDD) and substance use behaviors in the sex-combined group. (a) Forest plot of univariable MR results. (b) Forest plot of MVMR results adjusted for socioeconomic status (SES). Each line represents the beta estimate with its corresponding 95% confidence interval (CI), derived from different MR methods, distinguished by color and shape. Results are grouped by the direction of the causal inference. False discovery rate (FDR)-adjusted *p*-values are shown on the right for both univariable and MVMR estimates.

To provide an overall view of causal relationships between MDD and substance use traits, we conducted bidirectional two-sample MR using sex-combined GWAS data (Figure 3a, Supplementary Tables S6–S7). MDD showed positive causal effects on smoking initiation (IVW β = 0.31, SE = 0.051, p = 1.5 × 10^−9^) and PAU (IVW β = 0.09, SE = 0.030, p = 1.6 × 10^−3^), and a negative effect on drinking frequency (IVW β = −0.10, SE = 0.036, p = 4.2 × 10^−3^). These associations were consistent across all MR methods tested, including IVW, simple mode, weighted median, and weighted mode. However, no consistent evidence supported causal effects of substance use traits on MDD.

Sensitivity analyses supported the robustness of these findings. Although heterogeneity was detected by Cochran’s Q statistic and the MR-PRESSO global test, outlier-corrected estimates remained consistent with IVW results, and distortion *p*-values were non-significant through MR-PRESSO. Additionally, MR-Egger intercepts did not indicate directional pleiotropy, providing further support that horizontal pleiotropy did not materially bias the causal estimates.

We next performed multivariable MR (MVMR), including each socioeconomic status (SES) proxy as an additional exposure (Figure 3b). The MDD effects on smoking initiation and drinking frequency attenuated and became non-significant after SES adjustment of all three SES measurements, whereas the effect on PAU persisted and was significant across all measured SES proxies (Supplementary Tables S7–S9). In the reverse direction, SES-adjusted MVMR did not identify any significant effects of substance use traits on MDD (Supplementary Tables S10–S12).

### Bidirectional MR and MVMR analyses of MDD and substance use in females

**Figure 4.**
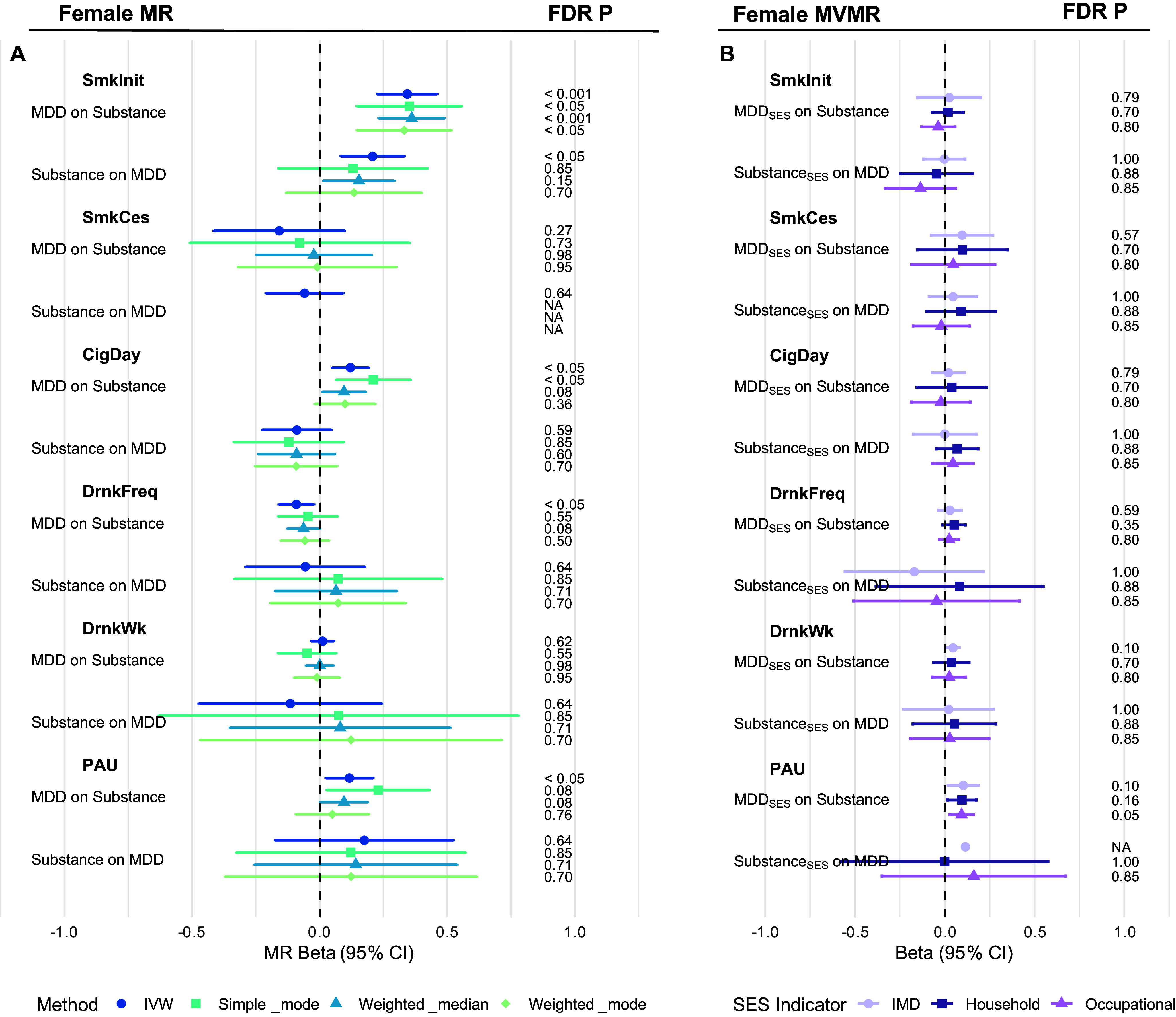
Forest plots of bidirectional MR and MVMR estimates of the causal relationship between MDD and substance use behaviors in females. (a) Forest plot of univariable MR results in females. (b) Forest plot of MVMR results adjusted for SES. Each line represents the beta estimate with its corresponding 95% confidence interval (CI), derived from different MR methods, distinguished by color and shape. Results are grouped by the direction of the causal inference. FDR-adjusted *p*-values are shown on the right for both univariable and MVMR estimates.

We next conducted sex-stratified bidirectional MR analyses in females to examine the causal relationships between MDD and substance use traits (Figure 4a; Supplementary Tables S6–S7). MDD showed a robust positive causal effect on smoking initiation (IVW β = 0.34, SE = 0.06, p = 9.6 × 10^−9^), with consistent results across all MR methods. MDD also showed a positive causal effect on PAU (IVW β = 0.12, SE = 0.05, p = 0.01), which remained significant after FDR correction. In the reverse direction, no substance use trait showed a consistently significant causal effect across methods.

For the MDD to smoking initiation analysis, Cochran’s Q and MR-PRESSO indicated heterogeneity. However, outlier correction with MR-PRESSO yields a similar estimate (β = 0.38, SE = 0.05, *p* = 1.32 × 10^−6^), suggesting minimal bias from horizontal pleiotropy.

In the MVMR adjusting for SES (Figure 4b), the effect of MDD on smoking initiation was completely attenuated, while the effect on PAU remained almost unchanged and was nominally significant across all SES proxies. Interestingly, the MDD effect on PAU adjusted for occupational income retained significance after FDR correction (Supplementary Tables S9–S11).

### Bidirectional MR and MVMR analyses of MDD and substance use in males

**Figure 5.**
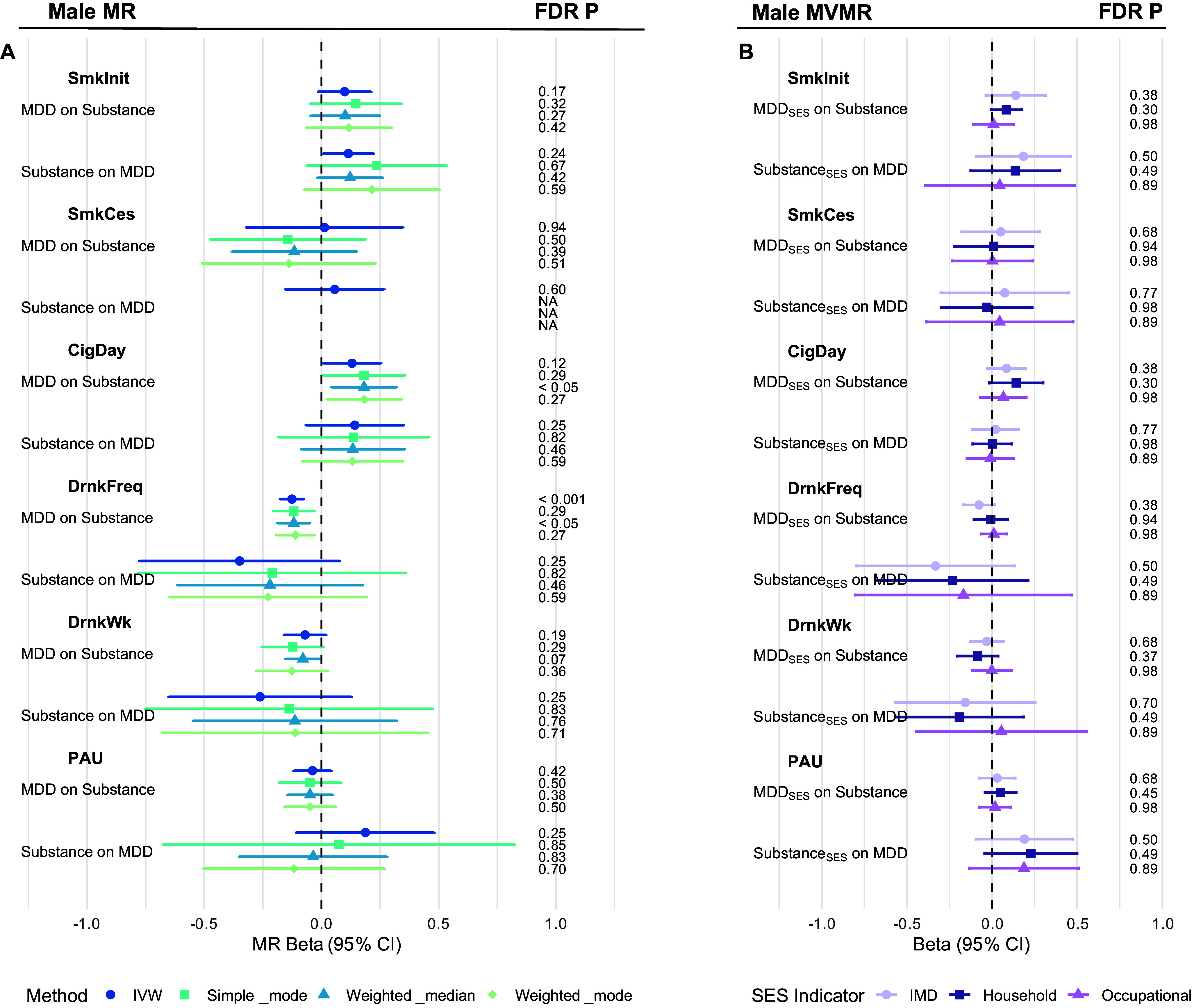
Forest plots of bidirectional MR and MVMR estimates of the causal relationship between MDD and substance use behaviors in males. (a) Forest plot of univariable MR results in males. (b) Forest plot of MVMR results adjusted for SES in males. Each line represents the beta estimate with its corresponding 95% confidence interval (CI), derived from different MR methods, distinguished by color and shape. Results are grouped by the direction of the causal inference. FDR-adjusted *p*-values are shown on the right for univariable and MVMR estimates.

Following the female-specific analyses, we conducted sex-stratified bidirectional MR in males (Figure 5a; Supplementary Tables S6–S7). Among all tested associations, only genetic liability to MDD showed a significant and consistent protective effect on drinking frequency (IVW β = −0.13, SE = 0.03, p = 9.6 × 10^−6^), suggesting that higher genetic risk for MDD was associated with less frequent alcohol consumption in males. Horizontal pleiotropy was not detected through Cochran’s Q, MR-PRESSO, or the MR-Egger intercept for the causal effect of MDD on drinking frequency.

The estimated effect of MDD on PAU in males was not statistically significant (IVW β = −0.04, SE = 0.04, p = 0.3). No other substance use traits showed consistent evidence of causal effects from MDD, nor did any trait show a significant causal effect on MDD in the reverse direction.

In the MVMR adjusting for SES (Figure 5b; Supplementary Tables S9–S11), the negative effect of MDD on drinking frequency was attenuated to non-significant for all three SES measures.

### Sensitive analyses

F-statistics were calculated to assess the strength of the genetic instruments used in the two-sample MR analyses. All exposures had mean F-statistics well above the conventional threshold of 10, indicating strong instruments and low risk of weak instrument bias (Supplementary Table S13). For the multivariable MR analyses, conditional F-statistics were generally above the conventional threshold for the primary exposures across all sex and substance traits (Supplementary Tables S7–S12). In addition, the MVMR Q-statistics suggested heterogeneity across most of the models. However, the SES-adjusted estimates were directionally consistent and did not alter the overall conclusions (Supplementary Tables S7–S12).

To verify the robustness of SNP-outcome associations after excluding UK Biobank data from the MDD GWAS, we compared the effect sizes of the instrumental variables in the full meta-analysis (with UKB) and the restricted dataset (excluding UKB). Effect sizes and *p*-values for these SNPs before and after UKB exclusion are provided in Supplementary Table S14. The estimates were highly correlated (Pearson’s r = 0.99 in females and 0.97 in males).

To specifically evaluate potential bias from sample overlap between the MDD and PAU, we applied the MRlap correction. MRlap estimates were broadly consistent with the estimates from the IVW method in both directions (Supplementary Table S15). For the significant causal effect from MDD to PAU in sex-combined and female groups, the MRlap corrected effects remained statistically significant and directionally consistent, although the magnitude was stronger after correction.

Given that variants in the *CHRNA5-A3-B4* gene cluster were included as instruments for CigDay, and *ADH1B* variants were used in the drinking traits, we repeated the analyses after removing these SNPs. The causal effects of CigDay and the drinking traits on MDD were retested following locus exclusion. The resulting estimates were directionally consistent and similar in magnitude to the main analyses, and no new causal effects emerged (Supplementary Table S16).

## Discussion

In this study, we conducted sex-stratified bidirectional MR and MVMR analyses to investigate the causal relationships between MDD and smoking and drinking. Genetic correlation analyses revealed significant shared genetic architecture between MDD and all substance use traits in the sex-combined GWAS. While patterns were broadly consistent across sexes, average weekly alcohol consumption was only correlated with MDD in females, and the correlation with cigarettes per day was significantly stronger in females.

In the sex-combined MR analyses, we observed that genetic liability to MDD was positively associated with smoking initiation and PAU. These findings are consistent with previous MR studies reporting causal effects of depression on smoking initiation (Wootton et al., 2020) and alcohol dependence (Polimanti et al., 2019). We also found that increased liability to MDD was associated with reduced alcohol drinking frequency. Although this relationship has not been reported in previous MR studies, it aligns with our observed negative genetic correlation between MDD and drinking frequency. In contrast, we found no consistent evidence that liability to smoking or alcohol use causally influenced risk for MDD, even though larger numbers of instruments were available for some traits.

In the sex-stratified MR, we found a significant causal effect of MDD on smoking initiation in females but not in males. Supporting this, a large epidemiological study found that early-initiating female smokers had significantly higher odds of MDD compared to males (Thompson, Tebes, & McKee, 2015), suggesting a stronger association between smoking and underlying depression in females. This may reflect differences in smoking motivation across sexes, females are more likely to initiate smoking in response to stress and depressive symptoms (Perkins et al., 2012), whereas males are more often driven by social or hedonic motives (Rodríguez-Bolaños et al., n.d.). Notably, the observed causal effect in females was attenuated after adjusting for SES. This is consistent with previous findings that lower SES is associated with a higher likelihood of smoking initiation (Wellman et al., 2018), and education is inversely associated with both depression and smoking (Schmitz, Gard, & Ware, 2019). We found no evidence for a causal effect in males, either before or after SES adjustment. This pattern is consistent with a sex-by-SES interaction analysis, which revealed that low SES was more strongly linked to earlier smoking initiation among women in the UK (Peters, Huxley, & Woodward, 2014).

Another trait that showed suggestive causal effects in females but not in males was problematic alcohol use (PAU). This finding is consistent with prior evidence indicating that depression more strongly predicts later alcohol dependence in women (Kessler et al., 1997). This sex difference may reflect a greater tendency among females to use alcohol as a coping strategy for negative affect, including depressive symptoms (Lau-Barraco, Skewes, & Stasiewicz, 2009), and greater vulnerability to developing and relapsing into alcohol use disorder in response to equivalent levels of stress (Becker & Koob, 2016; Verplaetse et al., 2018). Notably, adjusting for SES did not substantially change the causal effect of MDD on PAU in females. This is consistent with prior observational studies in the United States (Lasserre et al., 2022) and Norway (Martinez et al., 2015), which found that controlling for education did not attenuate associations between depression and alcohol use disorder. Unlike PAU, which remained unaffected by SES adjustment, we did not observe the causal effect of MDD on weekly alcohol consumption (DrnkWk) before and after adjusting for SES. Despite both PAU and DrnkWk capturing aspects of alcohol use, our findings suggest a separation between typical consumption and problematic use. This aligns with the modest genetic correlation between UKB-derived alcohol consumption and clinically defined alcohol use disorder (rg = 0.37) (J. D. White & Bierut, 2023). Furthermore, alcohol consumption in the UKB has been shown to be influenced by higher educational attainment in both sexes (T. Zhou et al., 2021). This suggests that alcohol consumption in the UKB may partly reflect socially patterned behaviors rather than harmful use.

We also observed that the increased liability of MDD will decrease drinking frequency in males only. Unlike PAU, which clearly reflects problematic alcohol use, drinking frequency in the UKB may not capture harmful use, as it shows no significant genetic correlation with alcohol use disorder (J. D. White & Bierut, 2023). Given that drinking in the UK is often viewed as a routine and expected part of social life, particularly in pubs or social gatherings (Morris et al., 2020), the negative association only observed in males may reflect sex differences in how depression impacts engagement in social drinking. As men are generally more likely to drink in public, while women tend to drink in more private contexts (Erol & Karpyak, 2015), the male-specific negative association may reflect depression-related reductions in social engagement (Kupferberg & Hasler, 2023). Since drinking frequency in the UKB likely reflects socially patterned behavior and has been linked to higher SES in both Swedish epidemiological (Heckley, Jarl, & Gerdtham, 2017) and UK-based MR studies (T. Zhou et al., 2021), adjusting for SES is critical when interpreting its relationship with depression.

This study demonstrates the importance of applying MR in a sex-stratified framework. By analyzing females and males separately, we uncovered causal effects that would have been obscured in sex-combined analyses. Our findings also emphasize the critical role of socioeconomic status as a confounder in the relationship between depression and substance use, with the confounding effects potentially differing by sex. Together, these findings underscore the importance of both sex-stratified analyses and careful adjustment for SES in clarifying the causal pathways linking depression and substance use.

Despite leveraging the largest available GWAS, multiple MR approaches, and sex-stratified analyses, our study is limited by the use of data from individuals of European ancestry only. Furthermore, several methodological limitations should be acknowledged. First, MR relies on key assumptions, and residual pleiotropy cannot be fully ruled out. Second, the number of available genetic instruments for depression was larger in females than in males, which complicates direct comparisons of causal estimates across sexes. However, when significant associations were observed in males but not females, such as drinking frequency, these are less likely to be explained by instrument number differences. Third, potential sample overlap, particularly between SES and substance use GWAS, may bias MVMR estimates. Finally, while depression and PAU data were derived from consortia-based meta-analyses, the other traits were based on UKB, where smoking and drinking behaviors are embedded in specific cultural norms. Patterns of substance use and their associations with depression and SES may differ in other settings, underscoring the need for future research in more diverse populations.

## Supporting information

10.1017/S0033291726103195.sm001 Hu et al. supplementary material 1 Hu et al. supplementary material

10.1017/S0033291726103195.sm002 Hu et al. supplementary material 2 Hu et al. supplementary material

## Data Availability

All scripts and UKB-based GWAS are available upon request.

## Supplementary material

The supplementary material for this article can be found at http://doi.org/10.1017/S0033291726103195.
