# Supplementary material for: Sex differences in the genetic and causal relationships between depression, smoking, and alcohol use: the role of socioeconomic status
Source: Psychol Med. 2026 Mar 2;56:e58. doi: 10.1017/S0033291726103195 (PMC12969216; doi:10.1017/S0033291726103195)

Supplementary Figure 1. Manhattan plots of smoking initiation (SmkInit) in females and males.


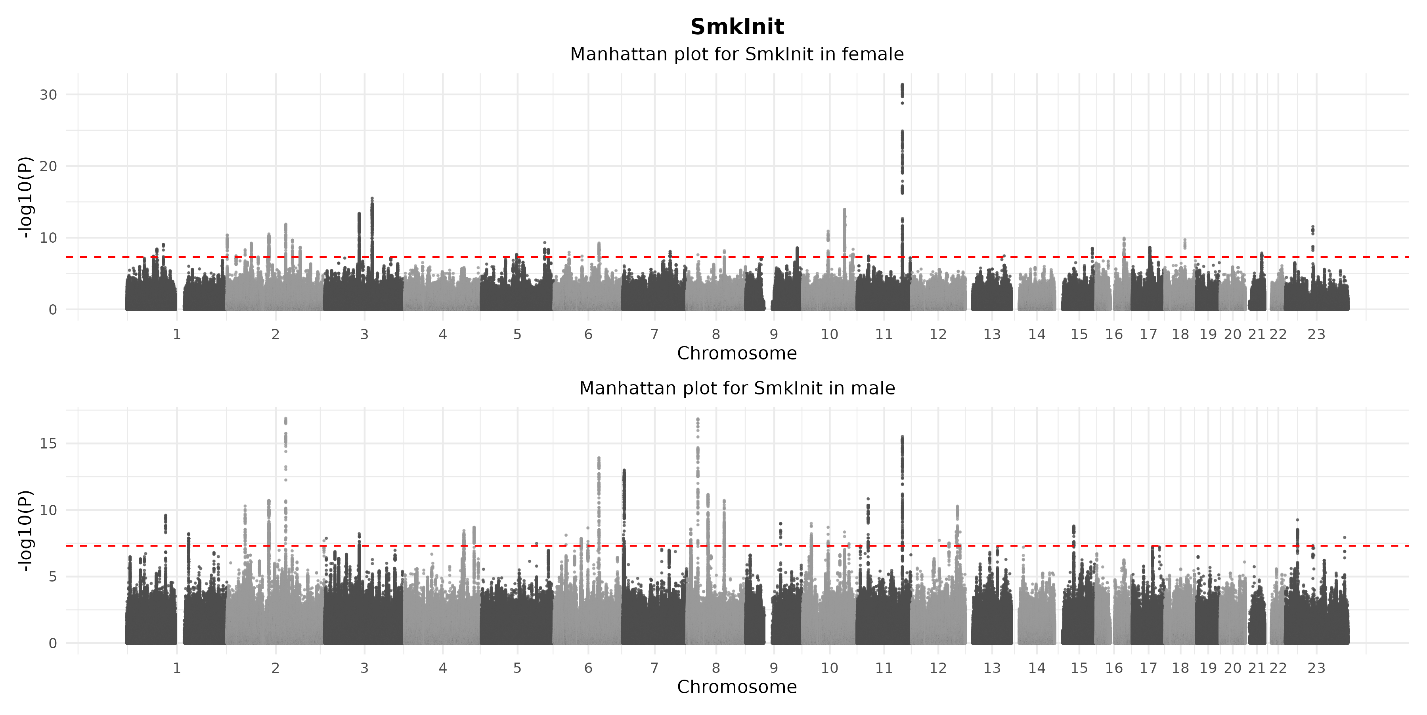


Supplementary Figure 2. Manhattan plots of smoking cessation (SmkCes) in females and males


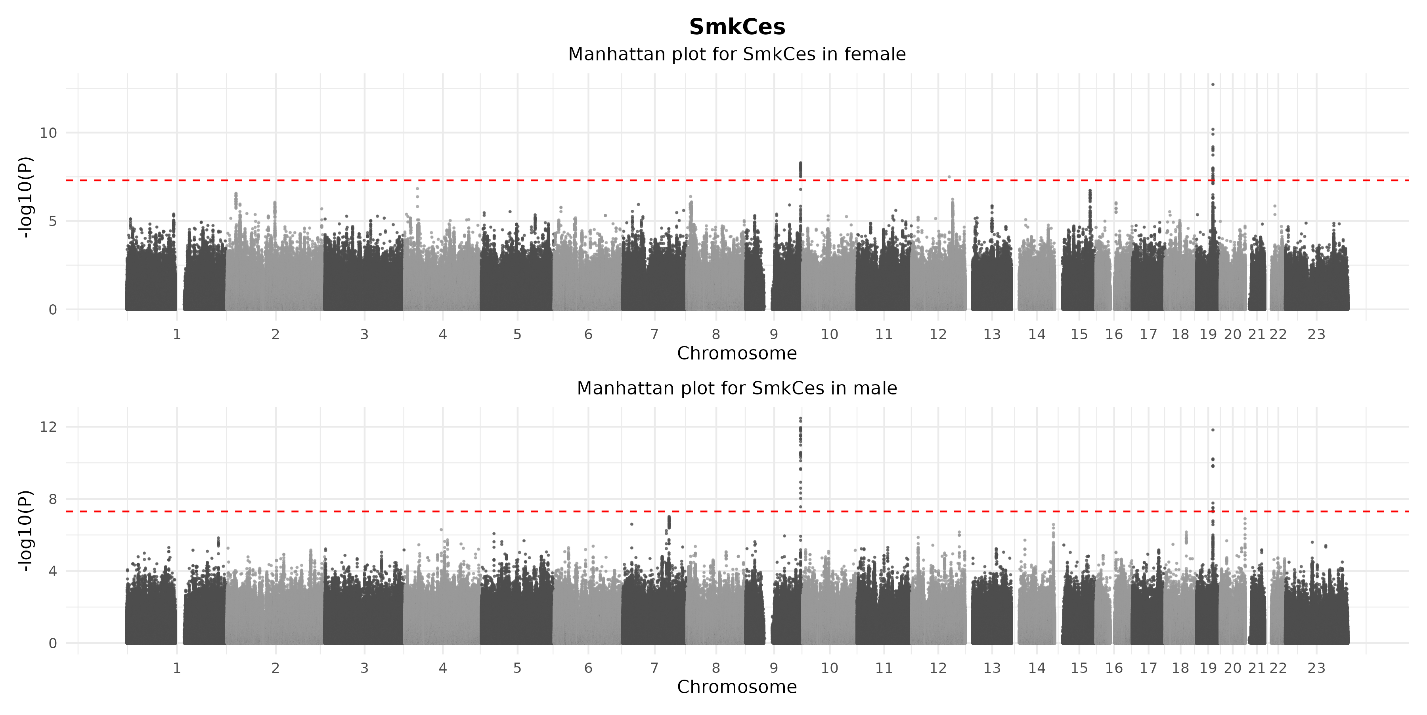


Supplementary Figure 3. Manhattan plots of cigarettes per day (CigDay) in females and males


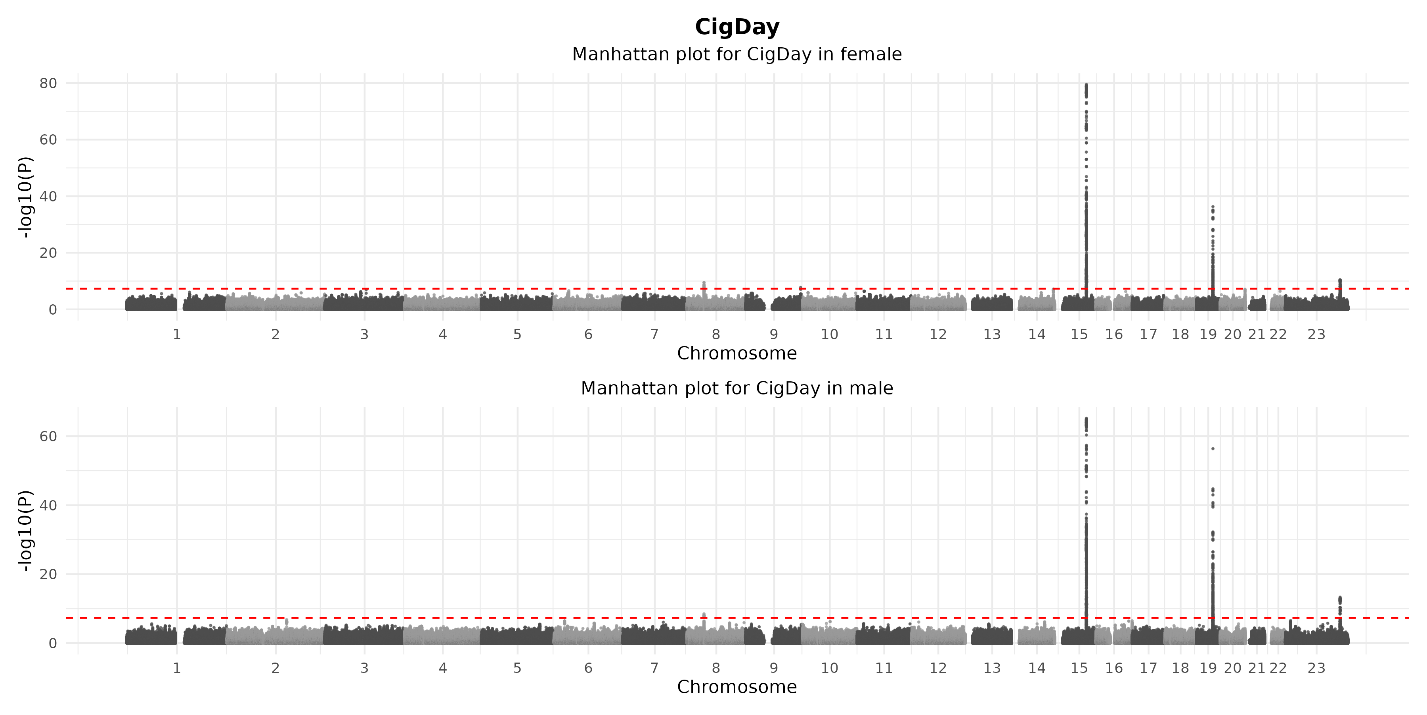


Supplementary Figure 4. Manhattan plots of drinks per week (DrnkWk) in females and males


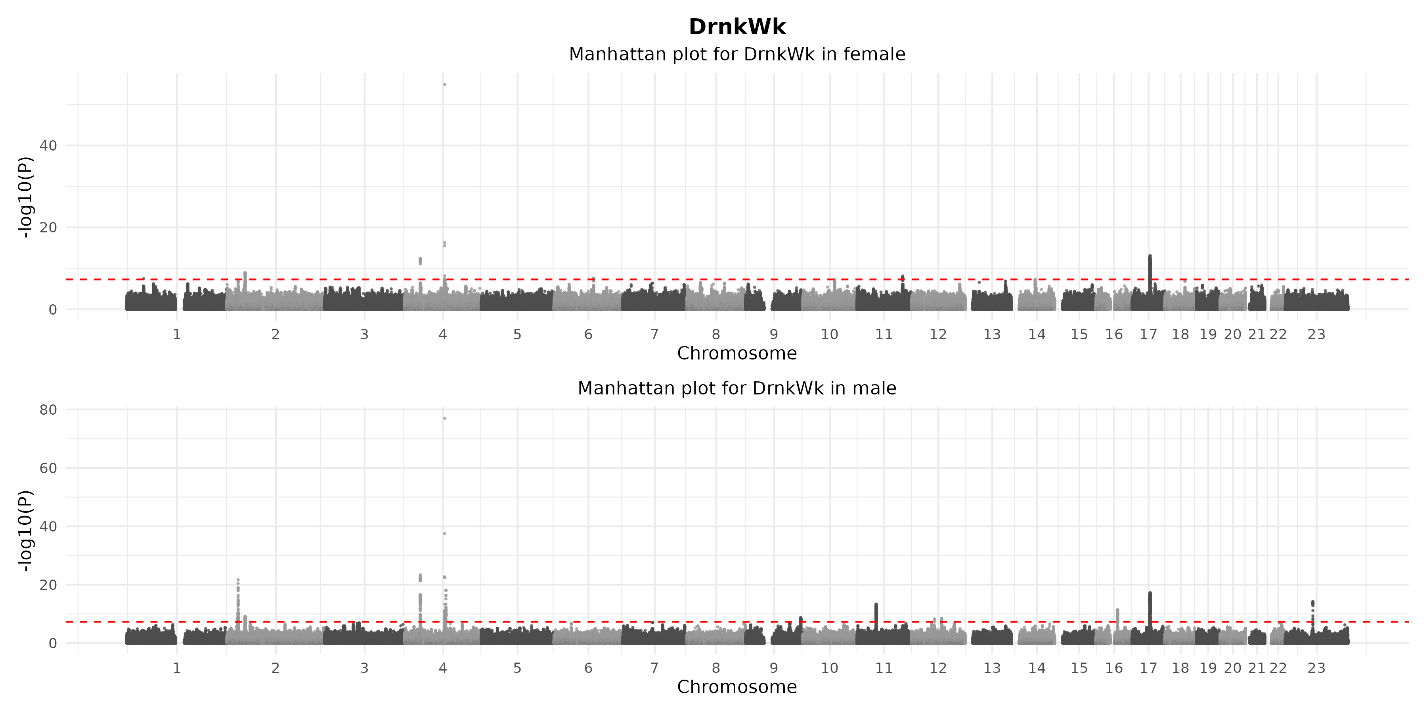


Supplementary Figure 5. Manhattan plots of drinking frequency (DrnkFreq) in females and males


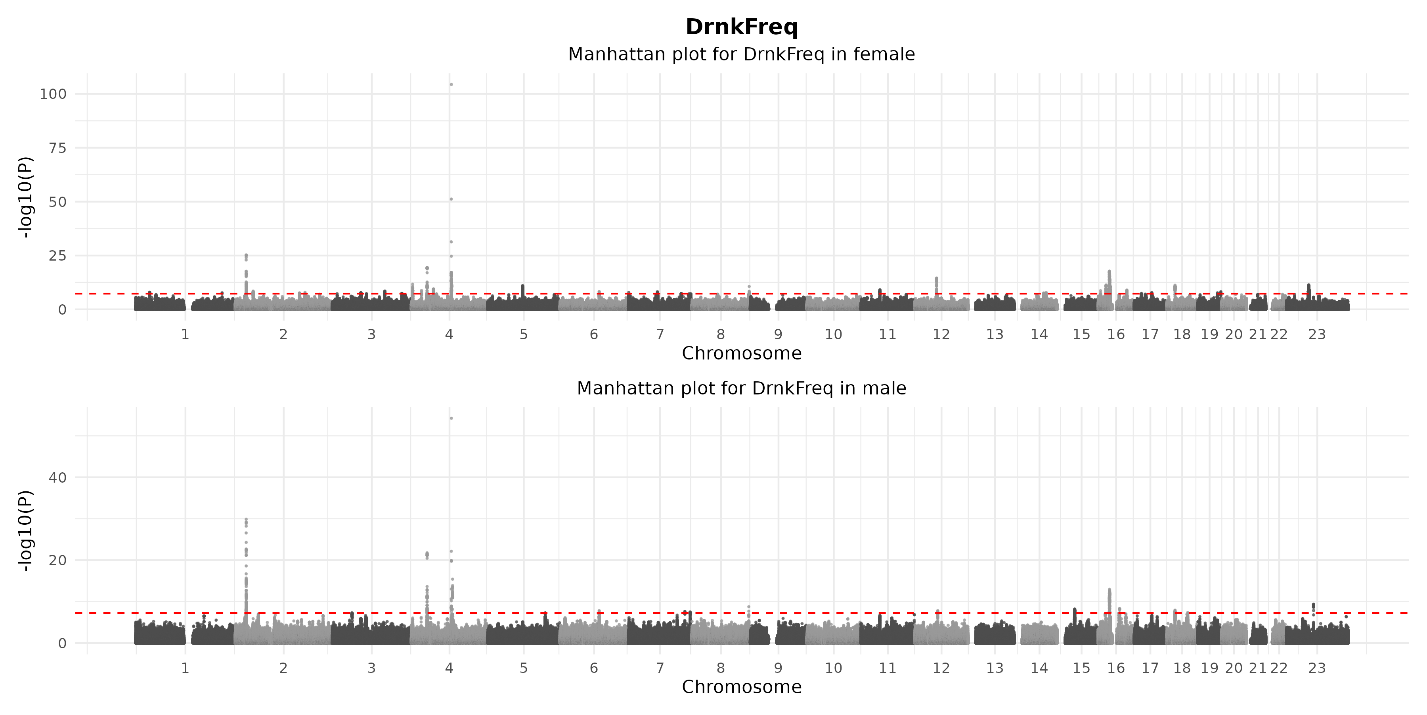


Supplementary Figure 6. Manhattan plots of socioeconomic status (SES) in females and males


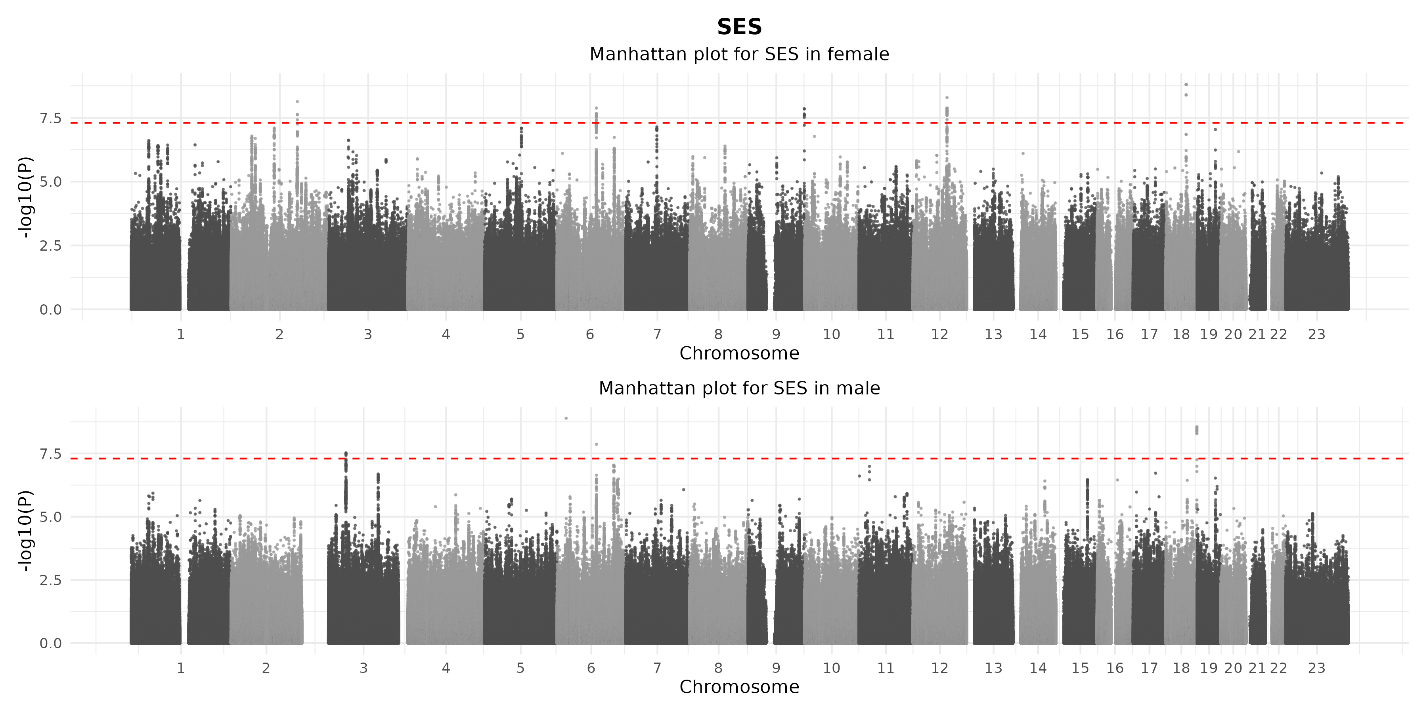

Supplement: Hu et al. supplementary material 1 — Hu et al. supplementary material [file S0033291726103195sup001.docx]
